# *Lactobacillus plantarum* Exhibits Antioxidant and Cytoprotective Activities in Porcine Intestinal Epithelial Cells Exposed to Hydrogen Peroxide

**DOI:** 10.1155/2021/8936907

**Published:** 2021-07-30

**Authors:** Jing Wang, Wei Zhang, Sixin Wang, Yamin Wang, Xu Chu, Haifeng Ji

**Affiliations:** ^1^Institute of Animal Husbandry and Veterinary Medicine, Beijing Academy of Agriculture and Forestry Sciences, 100097 Beijing, China; ^2^Sino-US Joint Laboratory of Animal Science, Beijing Academy of Agriculture and Forestry Sciences, 100097 Beijing, China; ^3^College of Agriculture, Qinghai University, 810016 Xining, China

## Abstract

Probiotics are widely used for protection against stress-induced intestinal dysfunction. Oxidative stress plays a critical role in gastrointestinal disorders. It is established that probiotics alleviate oxidative stress; however, the mechanism of action has not been elucidated. We developed an *in vitro* intestinal porcine epithelial cells (IPEC-J2) model of oxidative stress to explore the antioxidant effect and potential mode of action of *Lactobacillus plantarum* ZLP001. The IPEC-J2 cells were preincubated with and without *L. plantarum* ZLP001 for 3 h and then exposed to hydrogen peroxide (H_2_O_2_) for 4 h. Pretreatment with *L. plantarum* ZLP001 protected IPEC-J2 cells against H_2_O_2_-induced oxidative damage as indicated by cell viability assays and significantly alleviated apoptosis elicited by H_2_O_2_. *L. plantarum* ZLP001 pretreatment decreased reactive oxygen species production and the cellular malondialdehyde concentration and increased the mitochondrial membrane potential compared with H_2_O_2_ treatment alone, suggesting that *L. plantarum* ZLP001 promotes the maintenance of redox homeostasis in the cells. Furthermore, *L. plantarum* ZLP001 regulated the expression and generation of some antioxidant enzymes, thereby activating the antioxidant defense system. Treatment with *L. plantarum* ZLP001 led to nuclear erythroid 2-related factor 2 (Nrf2) enrichment in the nucleus compared with H_2_O_2_ treatment alone. Knockdown of Nrf2 significantly weakened the alleviating effect of *L. plantarum* ZLP001 on antioxidant stress in IPEC-J2 cells, suggesting that Nrf2 is involved in the antioxidative effect of *L. plantarum* ZLP001. Collectively, these results indicate that *L. plantarum* ZLP001 is a promising probiotic bacterium that can potentially alleviate oxidative stress.

## 1. Introduction

Oxidative stress is caused by an imbalance between prooxidants and antioxidants and is implicated in extensive human and animal diseases. Oxidative stress is often associated with the accumulation of reactive oxygen species (ROS), which can induce DNA hydroxylation, protein denaturation, and lipid peroxidation [1] and thus compromise the viability of cells, ultimately causing several diseases [2]. The intestine is more vulnerable to oxidative stress owing to its continuous exposure to the luminal environment. Intestinal oxidative stress influences the digestibility and absorption of nutrients and can cause various intestinal diseases [3, 4]. In particular, during the critical life phases of animals, such as weaning, the underdeveloped intestine combined with depressed intake can lead to the insufficient synthesis of endogenous antioxidants. Therefore, antioxidant supplementation strategies have been considered. Dietary antioxidants, such as vitamins C and E, and metals, such as Zn and Cu, can neutralize oxidative molecules and play an important role in maintaining redox homeostasis in humans and animals [5, 6].

Probiotic bacteria have been shown to exhibit antioxidant activity both *in vitro* and *in vivo* [7–9]. Several probiotic strains and their products present in food exert remarkable antioxidant activities, and these strains exhibit high viability in anaerobic environments, have a strong oxygen radical-scavenging ability, and produce several antioxidant enzymes [10–12]. Antioxidative properties vary widely among bacterial strains, indicating that they are strain specific [7, 13]. Probiotics have been demonstrated to exert antioxidant activities in various host cells and the human body by modulating the redox status by scavenging free radicals, chelating metal ions, regulating enzymes, and modulating the intestinal microbiota [8, 9]. Further, it has been reported that probiotics exert antioxidant activity mainly through the induction of detoxifying enzymes via the activation of transcription factor nuclear erythroid 2-related factor 2 (Nrf2) [14, 15]. Other regulatory pathways involving Sirt1, MAPK, and PKC, which may trigger the dissociation of Nrf2 or enhance the cell homeostasis, are also involved in the regulation of their antioxidant action [9]. However, several questions regarding the underlying mechanisms of the antioxidative roles of probiotics, such as concentration effects, mitochondrial function, and Nrf2 dissociation pattern, remain unsolved.

In our previous studies, we demonstrated that *L. plantarum* ZLP001 isolated from healthy piglet ileal mucosa [16] exerts a strong antioxidant ability, is highly viable in hydrogen peroxide, has a high oxygen radical-scavenging ability *in vitro*, and alleviates oxidative stress in weaning piglets *in vivo* [17]. However, the antioxidant capacity of *L. plantarum* ZLP001 under oxidative stress or its mechanisms of action is not well understood. Therefore, in this study, we evaluated the effect of *L. plantarum* ZLP001 pretreatment in an *in vitro* model of oxidative stress using porcine intestinal epithelial cells (IPEC-J2) treated with hydrogen peroxide (H_2_O_2_).

## 2. Materials and Methods

### 2.1. Bacterial Strain, Cells, and Culture Conditions

*L. plantarum* ZLP001 was originally isolated in our laboratory, from the ileal mucosa of healthy piglets 4 w after weaning. The strain was identified by the China Center of Industrial Culture Collection (Beijing, China) and is preserved in the China General Microbiological Culture Collection Center (CGMCC No. 7370). *L. plantarum* ZLP001 cells were cultured in improved de Man, Rogosa, and Sharpe liquid medium (10 g peptone, 5 g yeast powder, 20 g glucose, 10 g beef extract, 5 g sodium acetate, 2 g ammonium citrate dibasic, 2 g dipotassium phosphate, 0.58 g magnesium sulfate, 0.19 g manganese sulfate, 1 mL of Tween-80, and water to 1,000 mL; pH 6.5) at 37°C under anaerobic conditions.

The porcine intestinal epithelial cell line (IPEC-J2) was a generous gift from Dr. Glenn Zhang (Oklahoma State University, Stillwater, OK). The IPEC-J2 cells were cultured in DMEM/F12, a 1 : 1 mixture of Dulbecco's modified Eagle's medium and Ham's F-12 (Gibco™, Thermo Fisher Scientific, Waltham, MA, USA) supplemented with 10% fetal bovine serum (FBS; Gibco™), streptomycin (100 *μ*g/mL), penicillin (100 U/mL), and 1% ITS premix (5 *μ*g/mL insulin, 5 *μ*g/mL transferrin, 5 ng/mL selenium; ScienCell, San Diego, CA) at 37°C in a 5% CO_2_ and 95% air atmosphere with 90% humidity.

### 2.2. Oxidative Stress Model Establishment

An *in vitro* oxidative stress model was established by treating IPEC-J2 cells with H_2_O_2_. Cell viability was assessed using the methyl thiazolyl tetrazolium (MTT) assay. IPEC-J2 cells were seeded in 6-well tissue culture plates (Costar, Corning Inc., Corning, NY, USA) at 2.5 × 10^5^ cells/well and cultured overnight with 2 mL of complete culture medium. After treatment with H_2_O_2_ at final concentrations of 0, 250, 500, 750, 1000, 1250, 1500, and 1750 *μ*mol/L for 4 h, the cells were incubated with 5 mg/mL of MTT working solution at 37°C for 4 h. The absorbance at 490 nm was measured using a Multiskan FC instrument (Thermo Fisher Scientific, Waltham, MA, USA). The median lethal dose (LD50) of H_2_O_2_ was calculated by probability unit and the optimal concentration of H_2_O_2_ was selected to establish the IPEC-J2 cell-based oxidative stress model.

### 2.3. Cell Treatments

IPEC-J2 cells were seeded in 6-well tissue-culture plates at 2.5 × 10^5^ cells/well. After overnight culture, the complete culture medium was replaced with culture medium without antibiotics, and the cells were incubated with *L. plantarum* ZLP001 at 10^5^, 10^6^, 10^7^, 10^8^, or 10^9^ CFU/well for 2 h, 3 h, or 4 h. After the bacteria were washed away with PBS, the cells were cultured in a complete culture medium with or without H_2_O_2_ (optimal H_2_O_2_ concentration obtained from the above experiment) for 4 h. Cell viability was determined using the MTT assay.

To estimate the antioxidant effect of *L. plantarum* ZLP001 on IPEC-J2 cells, four treatments were designed: a control, a *L. plantarum* ZLP001 treatment (optimal concentration and incubation time based on the above experiment), a H_2_O_2_ treatment, and a *L. plantarum* ZLP001 pretreatment+H_2_O_2_ treatment. After overnight culture, IPEC-J2 cells were incubated with *L. plantarum* ZLP001 in a complete culture medium without antibiotics for 3 h. Then, the bacteria were washed away with PBS, and the cells were cultured in a complete culture medium with or without H_2_O_2_ for 4 h. In addition, IPEC-J2 cells were cultured in a complete culture medium without antibiotics for 3 h and then treated or not treated with H_2_O_2_ under the conditions described above as oxidative stress control and nontreated control, respectively.

### 2.4. Observation of Cell Morphology

Morphological changes in IPEC-J2 cells after the treatments were observed under an optical microscope (Olympus, Tokyo, Japan) at a magnification of 100x. Images were acquired using the cellSens Entry system (Olympus).

### 2.5. Detection of Cell Apoptosis and Necrosis

Apoptosis and necrosis in treated IPEC-J2 cells were detected using the Apoptosis and Necrosis Assay Kit (C1056) (Beyotime Biotechnology, Haimen, China). After the treatments, the cells were stained with Hoechst 33342 (10 ng/mL) and propidium iodide (PI, 10 ng/mL) at 4°C in the dark for 20 min, according to the manufacturer's instructions. Condensed or fragmented nuclei of apoptotic cells were visualized and photographed under an inverted fluorescence microscope (IX71, Olympus).

Apoptotic cells were also detected by flow cytometry using an Annexin V PE/7-AAD Assay Kit (CA1030) (Solarbio, Beijing, China). Immediately after the treatments, the cells were collected and resuspended in a binding buffer. One hundred microliters of cell suspension were mixed with 5 *μ*L of Annexin V/PE and incubated in the dark at room temperature for 5 min. After the addition of 10 *μ*L of 20 *μ*g/mL 7-AAD and 400 *μ*L of PBS, the cells were immediately analyzed by flow cytometry (FACScaliburTM, BD Biosciences, San Jose, CA, USA). Experiments were performed in triplicate.

### 2.6. Assessment of Intracellular ROS Generation

Intracellular ROS accumulation was measured using a commercial ROS detection kit (S0033S) (Beyotime Biotechnology, Haimen, China) with the green, fluorescent probe DCFH-DA (2′,7′-dichlorofluorescein diacetate). After the treatments, the culture medium was removed, the ROS indicator DCFH-DA (10 *μ*M) in fresh FBS-free medium was added, and the cells were incubated at 37°C for 30 min. The cells were visualized and photographed under a fluorescence microscope (IX71, Olympus). To quantify ROS production, the fluorescence intensity was measured using a fluorescence microplate reader (Tecan, Männedorf, Switzerland) at excitation/emission wavelengths of 525/610 nm. ROS levels are expressed as the percentage of treated cells compared to control cells. Experiments were performed in triplicate.

### 2.7. Measurement of the Mitochondrial Membrane Potential (MMP)

The Mitochondrial Membrane Potential Assay Kit (Beyotime Biotechnology, Haimen, China) with the membrane-permeable dye, JC-1, was used to detect mitochondrial depolarization in cells. After the treatments, the cells were incubated in JC-1 solution at 37°C for 15 min. The potential-dependent aggregation of JC-1 in the mitochondria (labeled with red fluorescence) and of the monomeric form of JC-1 in the cytosol after mitochondrial membrane depolarization (labeled with green fluorescence) were detected using flow cytometry. The MMP is reflected by the proportion of JC-1 aggregates and monomers. Experiments were performed in triplicate.

### 2.8. Determination of Lactate Dehydrogenase (LDH) and Malondialdehyde (MDA)

After the treatments, the cells were gently washed twice with PBS and lysed using RIPA Lysis Buffer (containing PMSF) (Solarbio, Beijing, China) for 10 min. The cells were centrifuged and 10,000 × *g* at 4°C for 10 min, and the supernatants were collected. Protein concentrations were determined using a bicinchoninic acid (BCA) protein assay (BCA Protein Assay Kit; Pierce, Madison, WI, USA). Then, the levels of LDH and MDA were determined using a LDH assay kit (A020-2) and MDA assay kit (A003-2) (Jiancheng, Nanjing, China). Experiments were performed in triplicate.

### 2.9. Determination of Glutathione and Oxidized Glutathione (GSH and GSSG) Concentrations

The cell sample collection and protein extraction and concentration evaluation were the same as above for the determination of LDH and MDA. Thereafter, the concentration of GSH and GSSG was determined using a GSH assay kit (A006-1-1) and GSSG assay kit (A061-2-1) (Jiancheng, Nanjing, China). Experiments were performed in triplicate.

### 2.10. Determination of T-AOC, T-SOD, CAT, and GSH-Px Activities

The total antioxidant capacity (T-AOC, A015-1-2) and the activities of total superoxide dismutase (T-SOD, A001-1-2), catalase (CAT, A007-1-1), and glutathione peroxidase (GSH-Px, A005-1-2) were determined using commercial assay kits (Jiancheng, Nanjing, China). After the treatments, the cells were lysed, and cellular protein concentrations were determined as above. Then, the T-SOD, CAT, and GSH-Px activities are determined according to the instructions of the manufacturer and expressed as U/mg protein. Experiments were performed in triplicate.

### 2.11. Establishment of Nrf2-Knockdown IPEC-J2 Cells

Porcine Nrf2 siRNA 5′-GCCCAUUGAUCUCUCUGAUTT-3′ (sense) and 5′-AUCAGAGAGAUCAAUGGGCTT-3′ (antisense) were synthesized at GenePharma Co. Ltd. (Shanghai, China). The IPEC-J2 cells were seeded into 6-well plates (2.5 × 10^5^ cells/well), cultured overnight, and transfected with siRNAs using Lipofectamine 2000 (Invitrogen, Carlsbad, CA, USA) according to the manufacturer's instructions.

### 2.12. Quantitative Reverse-Transcription Polymerase Chain Reaction (RT-qPCR)

Total RNA was extracted from treated cells using RNAzol reagent (Molecular Research Center, Cincinnati, OH, USA) according to the manufacturer's instructions. RNA concentrations were determined using a NanoDrop spectrophotometer (Thermo Fisher Scientific, Waltham, MA, USA). The RNA (1 *μ*g) was reverse-transcribed into cDNA using an iScript™ cDNA Synthesis Kit (Bio-Rad, Hercules, CA, USA) according to the manufacturer's instructions. The qPCRs were run using iTaqTM Universal SYBR Green Supermix (Bio-Rad) in a QuantStudio 3 Real-Time PCR System (Thermo Fisher Scientific, Waltham, MA, USA). Porcine-specific primers used in this study were referred from other references or designed with Primer 5.0, and the sequences are listed in Supplementary Table S1. Target gene expression was normalized to that of glyceraldehyde-3-phosphate dehydrogenase (*GAPDH*); relative fold changes in gene expression were calculated using the 2^–*ΔΔ*Ct^ method [18].

### 2.13. Western Blot

IPEC-J2 cells were collected after the treatments, and total proteins were extracted and protein concentrations were determined as above description. Equal amounts of protein (30 *μ*g) were loaded per lane and the protein was separated at 110 V for 1 h and then transferred to the PDVF membrane at 4°C and 90 V for 60-100 min. After blocking with 5% skim milk, the blots were incubated with primary antibodies at 4°C overnight. After three washes with Tris-buffered saline, the blots were incubated with an HRP-conjugated secondary antibody. Chemiluminescence detection was performed using Western Blot Luminance Reagent (Santa Cruz Biotechnology, Santa Cruz, CA, USA) according to the manufacturer's instructions. The antibodies used in this study are listed in Supplementary Table S2. Immunoreactive bands were imaged using the ChemiDoc XRS system (Bio-Rad) and were quantified using ImageJ (National Institutes of Health, Bethesda, MD, USA).

### 2.14. Statistical Analysis

All results are expressed as the mean ± standard error of the mean (SEM). Data analyses were performed using Prism version 6 (GraphPad Software, Inc., San Diego, CA, USA). Means were compared using one-way analysis of variance (ANOVA) followed by Duncan's *post hoc* tests in SPSS (version 20.0, SPSS Inc., Chicago, IL, USA). Means of two groups were compared using unpaired Student's two-tailed *t*-test. *P* < 0.05 was considered a significant difference.

## 3. Results

### 3.1. Establishment of an Oxidative Stress Model in IPEC-J2 Cells

**Figure 1:**
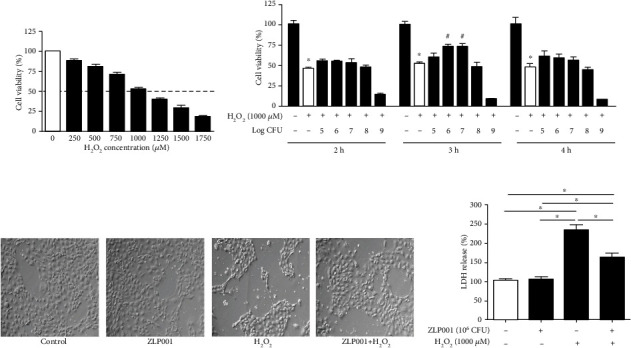
*Lactobacillus plantarum* ZLP001 protects IPEC-J2 cells against H_2_O_2_-induced oxidative stress damage. (a) H_2_O_2_-induced IPEC-J2 cell viability reduction. IPEC-J2 cells were incubated with the indicated concentrations of H_2_O_2_ for 4 h; after which, cell viability was measured by the methyl thiazolyl tetrazolium (MTT) assay. (b) *L. plantarum* ZLP001 protects against H_2_O_2_-induced cell damage. IPEC-J2 cells were incubated with or without *L. plantarum* ZLP001 at the indicated concentrations for 2 h, 3 h, or 4 h; after which, the medium was replaced with fresh medium containing 1,000 *μ*M H_2_O_2_. After incubation for 4 h, cell viability was measured by the MTT assay. (c) Morphological analysis of the protective effect of *L. plantarum* ZLP001 (10^6^ CFU, 3 h) against H_2_O_2_-induced IPEC-J2 cell damage by microscopy. (d) Lactate dehydrogenase (LDH) release after the treatments as determined using a LDH assay. All data represent the mean ± standard error of mean (SEM) of three independent experiments. Means were compared by one-way ANOVA followed by Duncan's *post hoc* tests. ^∗^*P* < 0.05. The means of the two groups were compared using Student's *t*-test. ^∗^*P* < 0.05 vs. nontreated control cells; ^#^*P* < 0.05 vs. H_2_O_2_-treated cells.

To establish the oxidative stress model in porcine IPEC-J2 cells, we used the MTT method to determine the cell viability of IPEC-J2 after treatment with H_2_O_2_. As shown in Figure 1(a), H_2_O_2_ dose-dependently decreased the viability of IPEC-J2 cells. After 4 h of treatment with 1,000 *μ*M H_2_O_2_, the cell viability of IPEC-J2 was reduced to 52.8% ± 4.7%. Therefore, this concentration of 1,000 *μ*M and 4 h treatment time were used to induce oxidative stress in subsequent experiments.

### 3.2. *L. plantarum* ZLP001 Attenuated the Cell Damage Caused by H_2_O_2_

As shown in Figure 1(b), a 3-h pretreatment with 10^6^ or 10^7^ CFU *L. plantarum* ZLP001 increased IPEC-J2 cell viability by approximately up to 70%-75% after H_2_O_2_ insult, indicating that *L. plantarum* ZLP001 attenuates H_2_O_2_-induced cell damage. Lower and higher concentrations of *L. plantarum* ZLP001 were less effective, while 10^9^ CFU *L. plantarum* ZLP001 decreased the viability of IPEC-J2 directly and did not protect against H_2_O_2_-induced damage. Pretreatment with *L. plantarum* ZLP001 for 3 h more effectively protected cell viability than 2 h or 4 h pretreatment. We selected 10^6^ CFU *L. plantarum* ZLP001 concentration and 3 h pretreatment time to proceed with further research.

Cell morphology after the different treatments was examined by optical microscopy (Figure 1(c)). Compared with normal cells, the gaps between H_2_O_2_-treated IPEC-J2 cells were enlarged, and the cell membrane showed a loose structure. After pretreatment with *L. plantarum* ZLP001, less damage to cell integrity was observed.

LDH is a soluble cytosolic enzyme that is released when the cell membrane is damaged. To confirm the protective effect of *L. plantarum* ZLP001 on IPEC-J2 cells, LDH leakage after H_2_O_2_ treatment was measured (Figure 1(d)). While H_2_O_2_-treated IPEC-J2 cells showed significant LDH release compared to nontreated cells, pretreatment with *L. plantarum* ZLP001 strongly reduced LDH leakage in the culture supernatant.

### 3.3. *L. plantarum* ZLP001 Alleviates H_2_O_2_-Induced Apoptosis and Necrosis in IPEC-J2 Cells

**Figure 2:**
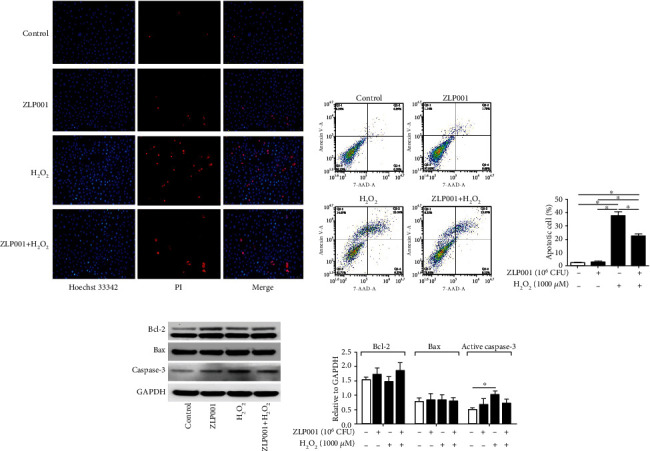
*Lactobacillus plantarum* ZLP001 inhibits H_2_O_2_-induced apoptosis in IPEC-J2 cells. IPEC-J2 cells were incubated with or without 10^6^ CFU *L. plantarum* ZLP001 for 3 h; after which, the medium was replaced with a fresh medium containing 1,000 *μ*M H_2_O_2_, and the cells were further incubated for 4 h. (a) Apoptosis detection based on Hoechst 33342 staining and PI staining. (b) Flow-cytometric analysis of apoptotic cells. (c) Quantification of apoptotic cells based on flow cytometry data. (d) Western blot analysis of Bcl-2, Bax, and active caspase-3 expression. (e) Quantitative analysis of apoptosis-related protein levels. All data represent the mean ± standard error of mean (SEM) of three independent experiments. Means were compared by one-way ANOVA followed by Duncan's *post hoc* test. ^∗^*P* < 0.05.

Hoechst 33342 and PI staining results showed that H_2_O_2_ considerably stimulated apoptosis (bright blue) and necrosis (bright red) in IPEC-J2 cells, indicating H_2_O_2_-induced oxidative damage. *L. plantarum* ZLP001 pretreatment significantly ameliorated H_2_O_2_-induced apoptosis and necrosis (Figure 2(a)). These findings were confirmed by flow cytometry results (Figures 2(b) and 2(c)).

The expression of the apoptosis-associated proteins, Bcl-2, Bax, and active caspase-3, was evaluated by western blot to further understand the antiapoptotic effect of the *L. plantarum* ZLP001 strain. Expression of the antiapoptotic factor Bcl-2 was enhanced by *L. plantarum* ZLP001 treatment, whereas no obvious increase was observed after H_2_O_2_ treatment alone (Figures 2(d) and 2(e)). After H_2_O_2_ treatment, activated caspase-3 expression was significantly increased, and this induction was alleviated by *L. plantarum* ZLP001 pretreatment. No significant effect on Bax expression was observed.

### 3.4. *L. plantarum* ZLP001 Regulates the Cellular Redox State in H_2_O_2_-Treated IPEC-J2 Cells

**Figure 3:**
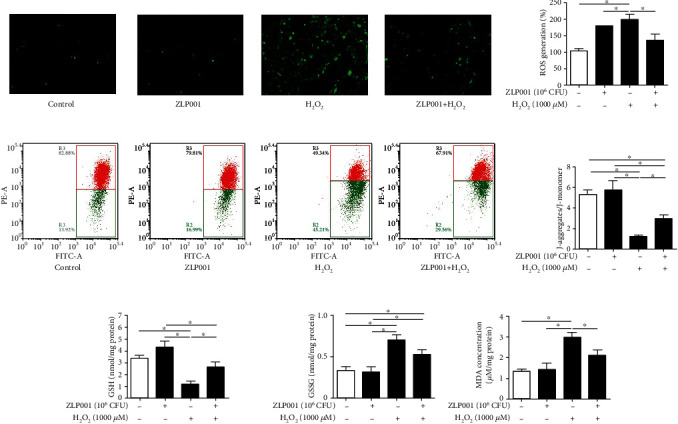
*Lactobacillus plantarum* ZLP001 improves the H_2_O_2_-induced redox state in IPEC-J2 cells. IPEC-J2 cells were incubated with or without 10^6^ CFU *L. plantarum* ZLP001 for 3 h; after which, the medium was replaced with a fresh medium containing 1,000 *μ*M H_2_O_2_, and the cells were further incubated for 4 h. (a) *L. plantarum* ZLP001 prevents H_2_O_2_-induced ROS accumulation in IPEC-J2 cells as indicated by DCFH-DA staining. (b) Quantification of the ROS levels based on DCFH-DA fluorescence. (c) Effect of *L. plantarum* ZLP001 on the mitochondrial membrane potential (MMP) in H_2_O_2_-induced IPEC-J2 cells as analyzed by flow cytometry. (d) Quantification of the effect on the MMP based on flow-cytometric data. (e) Concentrations of glutathione (GSH) and oxidized glutathione (GSSG) were determined using GSSG and GSH detection assays. (f) Concentrations of malondialdehyde (MDA) were determined using an MDA assay kit. All data represent the mean ± standard error of mean (SEM) of three independent experiments. Means were compared one-way ANOVA followed by Duncan's *post hoc* tests. ^∗^*P* < 0.05.

To evaluate the regulatory effect of *L. plantarum* ZLP001 on the H_2_O_2_-induced IPEC-J2 cell redox state, we measured intracellular ROS production using a cell-permeable, nonfluorescent probe DCFH-DA (Figures 3(a) and 3(b)). Unsurprisingly, ROS accumulation was significantly increased after exposure to H_2_O_2_, indicating that H_2_O_2_ caused an intracellular burst of ROS in IPEC-J2 cells. However, pretreatment of the cells with *L. plantarum* ZLP001 obviously suppressed the ROS burst induced by H_2_O_2_ in IPEC-J2 cells.

To further investigate the redox state, the MMP was determined (Figures 3(c) and 3(d)), which can be affected by H_2_O_2_-induced ROS release. After H_2_O_2_ treatment alone, the J-aggregate-to-J-monomer ratio in IPEC-J2 cells was obviously decreased. After *L. plantarum* ZLP001 pretreatment, this ratio was increased, demonstrating the positive effect of *L. plantarum* ZLP001 on the redox state in IPEC-J2 cells.

Next, we detected GSSG and GSH, which serve as important indicators of the cellular redox state (Figure 3(e)). After H_2_O_2_ treatment, intracellular GSSG levels were significantly increased, while GSH levels were markedly decreased (*P* < 0.05). However, pretreatment with *L. plantarum* ZLP001 showed a tendency to prevent the effects of H_2_O_2_ on both GSSG (*P* > 0.05) and GSH (*P* < 0.05). The GSSG and GSH levels in *L. plantarum* ZLP001-treated cells were similar to those in control cells (*P* > 0.05).

MDA is the main product of ROS-induced lipid peroxidation. As an excellent indicator of oxidative stress, we also measured the MDA levels in the treated IPEC-J2 cells. As shown in Figure 3(f), MDA levels in H_2_O_2_-treated IPEC-J2 cells were markedly increased compared to those in control cells, indicating the occurrence of lipid peroxidation. *L. plantarum* ZLP001 pretreatment significantly inhibited lipid peroxidation in IPEC-J2 cells, as indicated by the lower levels of MDA.

### 3.5. *L. plantarum* ZLP001 Upregulates the Antioxidant Defense System in H_2_O_2_-Treated IPEC-J2 Cells

**Figure 4:**
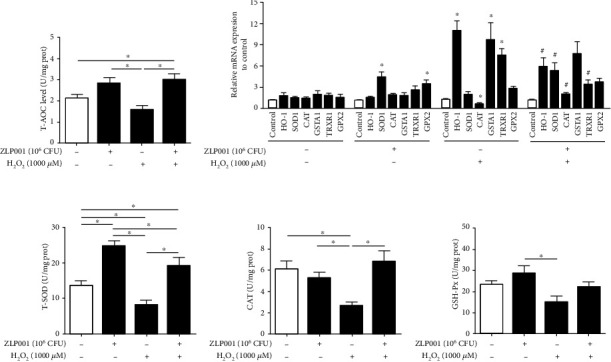
Effect of *Lactobacillus plantarum* ZLP001 on antioxidant enzyme expression and activity in IPEC-J2 cells. IPEC-J2 cells were incubated with 10^6^ CFU *L. plantarum* ZLP001 for 3 h; after which, the medium was replaced with a fresh medium containing 1,000 *μ*M H_2_O_2_, and the cells were further incubated for 4 h. (a) T-SOD activity in lysed cells was determined using a commercial assay kit. (b) The mRNA levels of *HO-1*, *SOD-1*, *CAT*, *GSTA1*, *TRXR1*, and *GPX2* as determined by RT-qPCR. (c–e) T-SOD, CAT, GSH-Px activities in lysed cells were determined using commercial assay kits. All data represent the mean ± standard error of mean (SEM) of three independent experiments. Differences between groups were analyzed by one-way ANOVA followed by Duncan's *post hoc* tests. ^∗^*P* < 0.05. Differences between two groups were determined by Student's *t*-test. ^∗^*P* < 0.05 vs. nontreated control cells; ^#^*P* < 0.05 vs. H_2_O_2_-treated cells.

The effect of *L. plantarum* ZLP001 on the antioxidant defense system in the cells was examined to further investigate how *L. plantarum* ZLP001 alleviated oxidative stress in the cells. The T-AOC activities in *L. plantarum* ZLP001-treated cells showed a numerically increase compared with the normal control group (Figure 4(a)). While the pretreatment of *L. plantarum* ZLP001 can significantly attenuate the decrease of T-AOC activity caused by H_2_O_2_.

As shown in Figure 4(b), the *HO-1*, *GSTA1*, and *TRXR1* mRNA expressions were significantly elevated in cell response to H_2_O_2_, whereas *CAT* expression was significantly decreased, indicating that the cells activated antioxidant mechanisms to protect themselves from oxidative stress. Compared with H_2_O_2_ treatment alone, *L. plantarum* ZLP001 pretreatment obviously suppressed *HO-1* and *TRXR1* mRNA expression, whereas it increased *SOD1* and *CAT* expression. *L. plantarum* ZLP001 treatment alone markedly elevated the mRNA expression of *SOD1* and *GPX2*, whereas no significant effects on other antioxidant enzymes were observed compared with control cells.

We further determined the activities of T-SOD, CAT, and GSH-Px using commercial assay kits (Figures 4(c)–4(e)). In line with the gene expression results, these enzyme activities treated or pretreated with *L. plantarum* ZLP001 were significantly regulated compared with control and H_2_O_2_ treatment alone. After *L. plantarum* ZLP001 pretreatment, the decline in antioxidant enzymes caused by H_2_O_2_ was alleviated. These results indicate that *L. plantarum* ZLP001 boosts endogenous antioxidant molecules. Collectively, the antioxidant effect of *L. plantarum* ZLP001 can be attributed to the activation of the antioxidant defense system.

### 3.6. *L. plantarum* ZLP001 Activates Nrf2 Signaling Pathway in IPEC-J2 Cells

**Figure 5:**
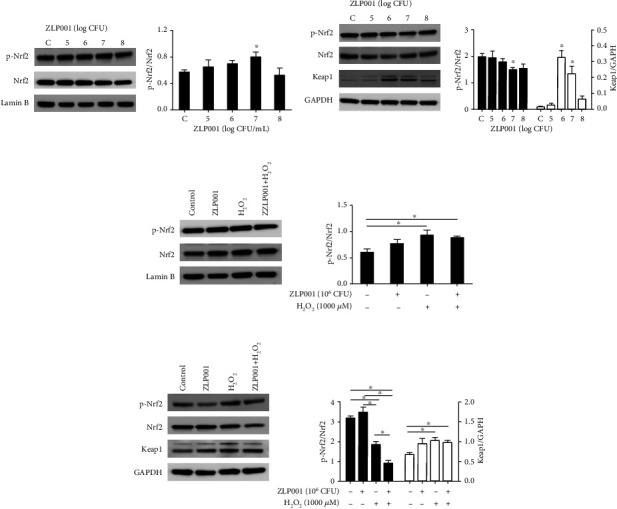
Effects of *Lactobacillus plantarum* ZLP001 on the Nrf2/Keap1 pathways in IPEC-J2 cells. (a) Protein levels of Nrf2 and p-Nrf2 as detected by western blot in nucleus (treated with different concentrations of *L. plantarum* ZLP001). (b) Protein levels of Nrf2, p-Nrf2, and Keap1 as detected by western blot in cytoplasm (treated with different concentrations of *L. plantarum* ZLP001). IPEC-J2 cells were incubated with indicated concentrations of *L. plantarum* ZLP001 for 3 h. After isolation of the nuclei from the cells, the nuclear Nrf2 and cytosolic Nrf2 and Keap1 were determined by immunoblotting. (c) Protein levels of Nrf2 and p-Nrf2 as detected by western blot in nucleus (treated with *L. plantarum* ZLP001 followed by H_2_O_2_). (d) Protein levels of Nrf2, p-Nrf2, and Keap1 as detected by western blot in cytoplasm (treated with *L. plantarum* ZLP001 followed by H_2_O_2_). IPEC-J2 cells were incubated with or without 10^6^ CFU *L. plantarum* ZLP001 for 3 h; after which, the medium was replaced with a fresh medium containing 1,000 *μ*M H_2_O_2_, and the cells were further incubated for 4 h. After isolation of the nuclei from the cells, the nuclear Nrf2 and cytosolic Nrf2 and Keap1 were determined by immunoblotting. All data represent the mean ± standard error of mean (SEM) of three independent experiments. Differences between the two groups were determined by Student's *t*-test. ^∗^*P* < 0.05 vs. nontreated control cells. Differences between groups were analyzed by one-way ANOVA followed by Duncan's *post hoc* tests. ^∗^*P* < 0.05.

To further explore the mode of action of *L. plantarum* ZLP001, we evaluated Nrf2 expression in IPEC-J2 cells treated with different concentrations of *L. plantarum* ZLP001 (Figures 5(a) and 5(b)). Immunoblotting results revealed that *L. plantarum* ZLP001 treatment dose-dependently decreased cytosolic Nrf2 and promoted nuclear Nrf2 accumulation, especially at 10^7^ CFU, indicating the occurrence of nuclear translocation of Nrf2. Cytosolic Keap1 accumulation was enhanced after *L. plantarum* ZLP001 treatment, especially at 10^6^ and 10^7^ CFU.

Next, we evaluated the translocation of Nrf2 after H_2_O_2_ treatment and pretreatment with *L. plantarum* ZLP001. As shown in Figures 5(c) and 5(d), H_2_O_2_ treatment alone had a remarkable effect on Nrf2 translocation from the cytosol to the nucleus. However, after pretreatment with *L. plantarum* ZLP001, the amount of Nrf2 in the cytosol showed a further decrease, while no significant accumulation of Nrf2 was observed in the nucleus compared with H_2_O_2_ treatment alone. The amount of Keap1 in the cytosol numerically increased after *L. plantarum* ZLP001 treatment alone, and significant differences were observed after H_2_O_2_ treatment alone and pretreatment with *L. plantarum* ZLP001.

### 3.7. Nrf2 siRNA Abolishes the Antioxidative Effects of *L. plantarum* ZLP001

**Figure 6:**
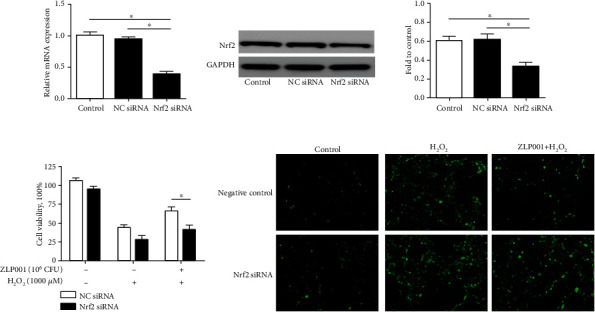
Effect of *Lactobacillus plantarum* ZLP001 on H_2_O_2_-induced oxidant stress in Nrf2-knockdown IPEC-J2 cells. IPEC-J2 cells were transfected with Nrf2 siRNA or negative control siRNA, and the efficiency of Nrf2 silencing was evaluated by RT-qPCR and western blot. (a) The mRNA level of *Nrf2*. (b) Western blot analysis of Nrf2 protein levels. Nrf2-knockdown and control cells were incubated with or without 10^6^ CFU *L. plantarum* ZLP001 for 3 h; after which, the medium was replaced with a fresh medium containing 1,000 *μ*M H_2_O_2_, and the cells were further incubated for 4 h. (c) Cell viability was measured using the methyl thiazolyl tetrazolium assay. The results were normalized to negative control siRNA-transfected cells without H_2_O_2_ and *L. plantarum* ZLP001 treatment. (d) ROS production was assessed by DCFH-DA staining and fluorescence microscopy. All data represent the mean ± standard error of mean (SEM) of three independent experiments. Differences between groups were analyzed by one-way ANOVA followed by Duncan's *post hoc* tests. ^∗^*P* < 0.05. Differences between the two groups were determined using Student's *t* test. ^∗^*P* < 0.05 vs. negative control siRNA-treated cells.

To further clarify the role of Nrf2 in the alleviation of oxidative stress by *L. plantarum* ZLP001, we evaluated cell viability and ROS production after Nrf2 knockdown in IPEC-J2 cells. Cells treated with Nrf2 siRNA effectively exhibited lower levels of Nrf2 than negative control siRNA-transfected cells and nontransfected control cells at both the gene and the protein level (Figures 6(a) and 6(b)). Nrf2 siRNA-transfected IPEC-J2 cells showed considerably lower cell viability than negative control cells when challenged with H_2_O_2_ (Figure 6(c)). The antioxidant-protective effect of *L. plantarum* ZLP001 was also blocked by Nrf2 siRNA, and the cells showed reduced viability when compared with negative control siRNA-transfected cells. Furthermore, Nrf2 knockdown weakened the effect of *L. plantarum* ZLP001 in preventing ROS generation in response to the H_2_O_2_ challenge (Figure 6(d)). These results demonstrate that Nrf2 siRNA abolishes the antioxidative action of *L. plantarum* ZLP001, suggesting that the Nrf2 pathway is involved in this action.

## 4. Discussion

An imbalance in the intestinal microbiota under stress or pathological conditions can result in the growth of pathogens, which may produce oxygen to generate an aerobic environment, thus rendering the intestine in an oxidative stress state [19, 20]. Intestinal oxidative stress causes damage to the epithelial barrier and affects nutrient digestibility and absorption and can lead to various diseases [3, 21, 22]. Thus, intestinal redox homeostasis is critical for maintaining host health. Although some probiotics have antioxidant capacity, the underlying mechanisms are not completely understood. In this study, we aimed to clarify the antioxidant capacity of *L. plantarum* ZLP001 under oxidative stress and its potential mechanism.

H_2_O_2_ is a strong oxidant capable of oxidizing a variety of moieties, which is why it is commonly used to establish an oxidative stress model to study redox-regulated processes in various cell types [23–25]. In the present study, MTT assays and the LD50 results demonstrated that treatment with 1,000 *μ*M H_2_O_2_ for 4 h was sufficient to induce oxidative stress in IPEC-2 cells. Pretreatment with *L. plantarum* ZLP001 (10^6^ CFU, 3 h) significantly alleviated the decrease of cell viability and release of LDH caused by H_2_O_2_, suggesting that *L. plantarum* ZLP001 has antioxidant capacity and can protect cell integrity. Previous studies have demonstrated that epithelial barrier injury is associated with oxidative stress [22, 26]. Our previous study revealed that *L. plantarum* ZLP001 has a positive effect on intestinal barrier function [27]; therefore, we speculate that the fortifying effect of *L. plantarum* ZLP001 on the intestinal barrier may be partially associated with its antioxidant ability. We used morphological analysis to observe nuclear condensation and DNA fragmentation, which are hallmarks of cell apoptosis [28, 29]. Apoptosis is generally induced when cells are subjected to oxidative stress [30, 31]. H_2_O_2_-treated IPEC-J2 cells showed obvious apoptosis, as demonstrated by the appearance of apoptotic nuclei based on Hoechst staining, compared with control cells. Pretreatment with *L. plantarum* ZLP001 obviously lowered the population of apoptotic nuclei in H_2_O_2_-induced IPEC-J2 cells. Flow cytometry results confirmed these findings. Probiotics have been previously demonstrated to have antiapoptotic effects and thus improve barrier function [32], suggesting they can indeed improve cell viability. Further, treatment with *L. plantarum* ZLP001 enhanced the expression of Bcl-2, while pretreatment suppressed the increase in activated caspase-3 caused by H_2_O_2_, corroborating the potential antiapoptotic role of *L. plantarum* ZLP001. Similar results were obtained by [14] using the probiotic strain *Bacillus amyloliquefaciens* SC06, although they only detected apoptosis-related gene expression and showed the image results. Taken together, our results indicate that *L. plantarum* ZLP001 has the potential to preserve intestinal integrity and barrier function under oxidative stress.

To confirm the antioxidant effect of *L. plantarum* ZLP001, we determined ROS production, MDA levels, and MMP. ROS are generated essentially for cellular growth and proliferation and have regulatory effects under physiological conditions [33–35]. Under normal conditions, intracellular ROS levels are maintained at a sustainable level. However, ROS overproduction, which occurs when ROS levels exceed the endogenous cellular capacity to remove them, will lead to oxidative damage of intracellular macromolecules, thus inducing a series of oxidative stress reactions [36]. H_2_O_2_ induced an intracellular ROS burst in IPEC-J2 cells as revealed by DCFH-DA staining. Pretreatment with *L. plantarum* ZLP001 remarkably reduced ROS accumulation induced by H_2_O_2_. The conversion of GSH to GSSG corroborated that *L. plantarum* ZLP001 could alleviate the oxidative stress caused by H_2_O_2_. GSH actively participates in scavenging ROS, but the conversion of GSH to GSSG after oxidation leads to the protein glutathionylation [37]. Levels of MDA, a product of lipid peroxidation, exhibited similar trends as the ROS levels in the present study. H_2_O_2_ treatment stimulated MDA secretion, whereas *L. plantarum* ZLP001 pretreatment inhibited the production of MDA induced by H_2_O_2_. As the largest contributors to intracellular oxidant production in most cell types, mitochondria are the site of major oxidative processes [38] and play a critical role in the oxidative stress-induced cell death [39]. We measured the MMP to evaluate the redox state of mitochondria. H_2_O_2_ decreased the MMP, whereas *L. plantarum* ZLP001 pretreatment enhanced the MMP. Mitochondrial dysfunction as indicated by a decrease in the MMP is considered a characteristic feature of early apoptosis [40]. The decrease in the MMP after H_2_O_2_ treatment in the present study suggested that apoptosis would be induced in the IPEC-J2 cells. Pretreatment with *L. plantarum* ZLP001 enhanced the MMP and markedly suppressed IPEC-J2 apoptosis. Thus, *L. plantarum* ZLP001 can effectively modulate the antioxidant status in IPEC-J2 cells. The limitation of our study is that the active molecules of this strain were not deeply studied. Studies have demonstrated that antioxidant molecules produced by probiotic strains, like exopolysaccharides and ferulic acid, may the key factors that probiotics play their antioxidant activity [41, 42]. Further isolation, purification, and structural elucidation may also need to evaluate the main active ingredients of *L. plantarum* ZLP001.

Enhancing the host antioxidant defense system leads to ROS scavenging in the body, thus alleviating oxidative stress. The protective effect of probiotics involves both nonenzymatic and enzymatic redox mechanisms [43]. To further investigate the antioxidant mechanisms of *L. plantarum* ZLP001, we evaluated T-AOC and mRNA expression and activities of some antioxidant-associated enzymes in IPEC-2 lysates. T-AOC normally reflects the capacity of the nonenzymatic antioxidant defense system [44] and is often used as a biomarker to investigate oxidative status [45]. The elevation in T-AOC after pretreatment with *L. plantarum* ZLP001 demonstrated that *L. plantarum* ZLP001 suppresses oxidative stress at least in part via the nonenzymatic antioxidant defense system. HO-1, a phase II enzyme, is transcriptionally regulated by various stimuli [46]. HO-1 is extremely sensitive to H_2_O_2_ induction and can be used as a sensitive target to screen antioxidants [14]. In the present study, we observed induction of *HO-1* gene expression by H_2_O_2_, which was suppressed by *L. plantarum* ZLP001 pretreatment. Antioxidant enzymes, such as SOD and CAT, can detoxify ROS to safe molecules, thus protecting cells against ROS damage [47, 48]. Increased expression of *SOD1* after *L. plantarum* ZLP001 treatment alone and increased *CAT* expression after *L. plantarum* ZLP001 pretreatment compared with H_2_O_2_ treatment was observed in the present study. Therefore, we also determined the expression levels of some other antioxidant-associated genes. *L. plantarum* ZLP001 treatment alone increased *GPX2* expression to elevate the antioxidant capacity. Similar results have been obtained in *Labeo rohita* fed probiotic and symbiotic diets [49]. The increase in *GSTA1* expression after H_2_O_2_ exposure may suggest a self-protective response to mitigate H_2_O_2_ toxicity [50]. Thioredoxin system activation was also observed after H_2_O_2_ treatment, and *L. plantarum* ZLP001 pretreatment decreased *TRXR1* expression, which is consistent with findings for several *Bacillus* strains research [14].

To confirm that *L. plantarum* ZLP001 enhanced the antioxidant system in IPEC-J2 cells, we measured antioxidase activity in IPEC-J2 cell lysates. In line with the gene expression results, *L. plantarum* ZLP001 increased T-SOD activity, and *L. plantarum* ZLP001 pretreatment alleviated the suppression of these enzyme activities by H_2_O_2_. The regulatory effects of probiotic strains on host antioxidative enzymes have been widely verified *in vitro* (in various cell models) and *in vivo* (in serum and in diverse tissues) [43, 50–52]. Our findings suggest that *L. plantarum* ZLP001 can dramatically improve the antioxidant status in IPEC-J2 cells by promoting cellular antioxidant defense systems against species generating ROS, thus rendering cells more resistant to the H_2_O_2_ challenge.

As the key endogenous pathway regulating the antioxidant system, activation of the Nrf2/Keap1-antioxidant response element (ARE) axis increases the transcription of antioxidant response elements, thus protecting cells and tissues against ROS damage [53–55]. The altered phase II gene expression and antioxidase activities imply that *L. plantarum* ZLP001 may affect the Nrf2/Keap1 pathway. Therefore, we investigated whether the Nrf2/Keap1-ARE pathway was involved in the antioxidant function of *L. plantarum* ZLP001. The expression of Nrf2/Keap1 pathway-related proteins in IPEC-J2 cells was enhanced by *L. plantarum* ZLP001, although differences were only significant at 10^7^ CFU. Previous studies have also revealed that probiotic administration in high fat diet-fed mice can induce Nrf2 expression compared with the control, and probiotic treatment alone *in vitro* was able to induce Nrf2 phosphorylation in IPEC-1 cells [14, 56]. However, as different probiotic strains induce variable levels of Nrf2 expression [57], we plan to conduct a comparative study of multiple strains and to investigate other potential pathways. Nrf2 is normally activated when cells are exposed to oxidants and electrophiles, thus protecting the cells against oxidative stress [54]. H_2_O_2_ stress increased Nrf2 phosphorylation in the present study, which concurs with Wang et al. [14]. After pretreatment with *L. plantarum* ZLP001, we observed enhanced dissociation of Nrf2 in the cytosol, but it did not accumulate in the nucleus compared to H_2_O_2_ treatment alone. Kobayashi and Yamamoto [58] reported that a phase II-activated defense system makes cells more resistant to subsequent even greater challenges. Thus, it is possible that *L. plantarum* ZLP001 may have activated Nrf2 before our detection time point, which made the IPEC-J2 cells more resistant to the subsequent challenge with H_2_O_2_ without a need for the continued accumulation of Nrf2. We further evaluated the cell viability, ROS production, and mRNA expression levels of antioxidative enzymes after Nrf2 knockdown in IPEC-J2 cells. The protective effect of *L. plantarum* ZLP001 on H_2_O_2_-induced IPEC-J2 cell damage was abolished under Nrf2 deficiency. These results confirm that the Nrf2-ARE signaling pathway is involved in the protective effect of *L. plantarum* ZLP001 and regulates the expression and activity of antioxidative enzymes to strengthen the defense against H_2_O_2_-induced oxidative stress in IPEC-J2 cells. Similarly, previous studies in different cells and hosts have reported that probiotic strains attenuate oxidative stress by upregulating Nrf2 expression, and by increasing the expression of antioxidative and cytoprotective genes [14, 15, 59]. However, Nrf2 activation by *L. plantarum* ZLP001 was not as significant as we expected and was not consistent with the antioxidant activity. As we have mentioned, other signaling pathways may also be involved in antioxidant regulation of *L. plantarum* ZLP001. Probiotics can attenuate hepatic oxidative stress via activating SIRT1 signaling [60] and ameliorate H_2_O_2_-induced epithelial barrier disruption through a PKC- and MAPK-dependent mechanism [61]. Whether these pathways were contributed to the antioxidant activity of *L. plantarum* ZLP001 is unknown. Thus, more robust, in-depth studies are required to unravel the precise antioxidant mechanism of *L. plantarum* ZLP001.

## 5. Conclusions

**Figure 7:**
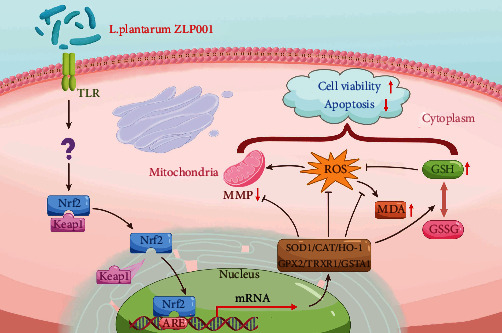
Suggested antioxidant protection mechanism of *Lactobacillus plantarum* ZLP001. *L. plantarum* ZLP001 may activate the Keap1-Nrf2 complex; after which, dissociated Nrf2 translocates to the nucleus and activates the transcription of cytoprotective genes, regulating the cellular redox state and activating the antioxidant defense system, thus alleviating cell apoptosis and necrosis caused by H_2_O_2_-induced oxidative stress.

We demonstrated that *L. plantarum* ZLP001 can alleviate oxidative damage induced by H_2_O_2_ in porcine epithelial cells. Pretreatment with *L. plantarum* ZLP001 alleviated H_2_O_2_-induced cell oxidative damage by regulating the redox state of cells and enhancing the antioxidant defense system, and Nrf2/Keap1-ARE signaling is involved in its protective mechanisms (Figure 7). Our results improve our understanding of the underlying mechanism of *L. plantarum* ZLP001 in protecting against oxidative stress, and it can be developed into a therapeutic or protective treatment for animals under oxidative stress. However, whether Nrf2 signaling is activated at an earlier time point as we speculated and which signaling triggered the dissociation of Nrf2 from its constitutive inhibitor, Keap1, or other potential pathways contribute to its antioxidation remain to be investigated more extensively.

## Data Availability

The data generated during the present study are available from the corresponding authors upon request.

## Supplementary Materials

Supplementary Materials Supplementary Table S1: sequences of the primers used in this study. Supplementary Table S2: antibodies used in this study.
