# Supplementary material for: Lactobacillus plantarum Exhibits Antioxidant and Cytoprotective Activities in Porcine Intestinal Epithelial Cells Exposed to Hydrogen Peroxide
Source: Oxid Med Cell Longev. 2021 Jul 30;2021:8936907. doi: 10.1155/2021/8936907 (PMC8349292; doi:10.1155/2021/8936907)
Supplement: Supplementary Materials — Supplementary Table S1: sequences of the primers used in this study. Supplementary Table S2: antibodies used in this study. [file 8936907.f1.zip › 8936907.f1/Supplementary Table S1.docx]

**Supplementary Table S1:** Sequences of the primers used in this study.

| Gene | Sequence | Product size (bp) | Accession number |
| --- | --- | --- | --- |
| *GAPDH* | F: 5′-GCTACACTGAGGACCAGGTTG-3′  R: 5′-CCTGTTGCTGTAGCCAAATTC-3′ | 146 | XM_021091114.1 |
| *SOD1* | F: 5′-GAGACCTGGGCAATGTGACT-3′  R: 5′-CTGCCCAAGTCATCTGGTTT-3′ | 139 | NM_001190422.1 |
| *CAT* | F: 5′- GGACATGGTCTGGGACTTCT-3′  R: 5′- GTCTTGCTGCATCTTCAACG-3′ | 221 | NM_214301.2 |
| *GSTA1* | F: 5′-CCGAGGCAGAATGGAGTGTA-3′  R: 5′-TGGTGGCGATGTAGTTGAGG-3′ | 197 | NM_214389.2 |
| *TRXR1* | F: 5′- CGGTCATCTCAGGGCACTCT-3′  R: 5′- TTCGTTTCCTTCTCCCACTT-3′ | 127 | NM_214154.3 |
| *GPX2* | F: 5′-GGCAGTGCTGATTGAGAATGT-3′  R: 5′-CAGGTAGGCGAAGACAGGAT-3′ | 271 | NM_001115136.1 |
| *HO-1* | F: 5′-TACCGCTCCCGAATGAACAC-3′  R: 5′-GTCACGGGAGTGGAGTCTTG-3′ | 209 | NM_001004027.1 |
| *Nrf2* | F: 5′-GAAAGCCCAGTCTTCATTGC-3′  R: 5′-TTGGAACCGTGCTAGTCTCA-3′ | 190 | XM_013984303.2 |
