# Supplementary material for: Lactobacillus plantarum Exhibits Antioxidant and Cytoprotective Activities in Porcine Intestinal Epithelial Cells Exposed to Hydrogen Peroxide
Source: Oxid Med Cell Longev. 2021 Jul 30;2021:8936907. doi: 10.1155/2021/8936907 (PMC8349292; doi:10.1155/2021/8936907)
Supplement: Supplementary Materials — Supplementary Table S1: sequences of the primers used in this study. Supplementary Table S2: antibodies used in this study. [file 8936907.f1.zip › 8936907.f1/Supplementary Table S2.docx]

**Supplementary Table S2:** Antibodies used in this study.

| Antibody | Supplier | Dilution |
| --- | --- | --- |
| Bax (cat no: #2772) | CST (Danvers, MA, USA) | 1:500 |
| Bcl-2 (#4223) | CST | 1:500 |
| Caspase 3 (#ab49822) | Abcam (Cambridge, MA, US) | 1:500 |
| GAPDH (#REK0005) | Real-Ab (Tianjing, China) | 1:5000 |
| Nrf2 (#ab92946) | Abcam | 1:500 |
| p-Nrf2 (#ab76026) | Abcam | 1:500 |
| Lamin B (#REK0017) | Real-Ab | 1:5000 |
| Keap1 (#ab118285) | Abcam | 1:500 |
